# Outcomes of Uniportal Video-Assisted Thoracoscopic Surgery in the Management of Lobectomy and Segmentectomy for Lung Cancer: A Systematic Review and Meta-Analysis of Propensity Score-Matched Cohorts

**DOI:** 10.5761/atcs.ra.24-00137

**Published:** 2025-03-01

**Authors:** I Wayan Sudarma, Putu Febry Krisna Pertiwi, Ketut Putu Yasa, I Komang Adhi Parama Harta

**Affiliations:** 1 Cardiothoracic and Vascular Surgery Division, Department of Surgery, Faculty of Medicine, Udayana University, Bali, Indonesia; 2 Faculty of Medicine, Udayana University, Bali, Indonesia

**Keywords:** lung neoplasm, thoracic surgery, video-assisted thoracoscopic surgery, survival analysis

## Abstract

**Purpose:** Uniportal video-assisted thoracoscopic surgery (UVATS) has been increasingly adopted for lung cancer management. This study aims to compare the perioperative and oncological outcomes of UVATS versus multiportal VATS (MVATS).

**Methods:** A comprehensive search was conducted on electronic databases. Perioperative outcomes evaluated were postoperative complications, conversion to open thoracotomy, and visual analog scale (VAS) scores on postoperative days 1 (POD1) and 3 (POD3). The oncological outcomes assessed were total lymph nodes retrieved. Individual patient time-to-event data were estimated from published Kaplan–Meier curves.

**Results:** The analysis demonstrated that UVATS was associated with significantly lower postoperative complications (relative risk [RR]: 0.76; 95% confidence interval [CI]: 1.64–0.91; *p* = 0.002), lower VAS scores on POD1(MD: –0.44; 95% CI: –0.70, –0.17; *p* = 0.001) and POD3 (MD: 0.76; 95% CI: –1.17, –0.36; *p* <0.001) compared to MVATS. Although UVATS had a lower conversion rate, this difference was not statistically significant (RR: 0.63; 95% CI: 0.33–1.18; *p* = 0.15). MVATS retrieved a higher number of lymph nodes, but this difference was also not statistically significant (MD: 0.6; 95% CI: –1.39, 0.12, *p* = 0.1). The overall survival probability at 96 months was slightly higher in the MVATS group (82.49%) compared to the UVATS group (75.89%), with a *p*-value of 0.5. Disease-free survival was comparable between the groups (75.43% UVATS and 74.74% MVATS, *p* = 0.59).

**Conclusion:** UVATS demonstrated favorable perioperative outcomes and comparable oncological efficacy to MVATS in the management of lobectomy and segmentectomy for lung cancer.

## Introduction

Surgical procedures are still the standard of care for lung cancer, especially in the early stages. Minimally invasive surgical techniques with video-assisted thoracoscopic surgery (VATS) for anatomical resection have been shown to provide benefits. The National Comprehensive Cancer Network (NCCN) recommends VATS for NSCLC patients without surgical contraindications. VATS also has been shown to provide better perioperative outcomes compared to conventional open thoracotomy procedures.^1)^ Conventionally, VATS uses a 3- to 4-port approach referred to as multiport VATS (MVATS), with one port serving for visualization and an additional port for instrumentation.^2)^ Subsequently, VATS evolved into a single-port approach, referred to as uniport VATS (UVATS). The uniport procedure was first published in 2000. In 2010, Gonzalez et al.^3)^ first developed the uniport VATS technique in NSCLC lobectomy. Since then, uniport VATS has been widely developed. Single-port VATS also has satisfactory results in complex lung resections involving segmentectomy, pneumonectomy, and bronchoplasty.^4)^ However, which one provides more benefits for patients, whether uniport or multiport is still a matter of debate. Several studies said uniport VATS can provide the same outcome effects as VATS with comparable results, with some mentioning that uniport VATS provides better effects compared to multiport VATS. However, not many studies have discussed the advantages of uniport VATS in terms of long-term oncological outcomes, such as overall survival and disease-free survival rates in lung cancer. Therefore, this study aims to (1) compare perioperative outcomes and (2) compare overall survival and disease-free survival between uniport and multiport VATS in the management of lobectomy and segmentectomy lung cancer.

## Materials and Methods

### Review design

This systematic review and meta-analysis were conducted under the Preferred Reporting Items for Systematic Reviews and Meta-analyses (PRISMA) guideline for ensuring transparency and quality in reporting.^5)^ This study has been registered on the PROSPERO CRD42024516844.

### Literature search and study selection

A literature search was conducted on PubMed, ScienceDirect, and Cochrane Library with Medical Subject Headings terms and keywords until 27 December 2023. The study focused on propensity score match (PSM) cohort studies (prospective and retrospective) reporting outcomes of single VATS procedures versus multiport VATS procedures in lobectomy and segmentectomy patients with early-stage lung cancer. The keywords used are as follows: ([Lung cancer] OR [Lung neoplasm]) AND ([video-assisted thoracoscopic surgery] OR [VATS] OR [thoracoscopic]) AND (uniport) AND ([multiport] OR [m-VATS] OR [t-VATS] OR [biportal] OR [bi-VATS]). The Population, Intervention, Comparator, Outcomes (PICO) method was applied in this study. The population consisted of patients with early-stage lung cancer patients who need lobectomy and or segmentectomy treatment. The inclusion criteria were lung neoplasm, peripheral lung cancer, clinical staging of T1–T3 and N0–N2, and no distant metastases, according to the 8th edition tumor, nodes and metastasis (TNM) classification.^6)^ The exclusion criteria were benign lung lesions; cT3 tumors invading the chest wall, diaphragm, or pericardium; sub-pulmonary lobectomy; and preoperative neoadjuvant therapy. The intervention group consisted of single or uniport VATS and the control group was conventional or multiport VATS.

### Outcomes of interest

The outcomes sought in this study were perioperative and oncological. Perioperative outcomes in this study included postoperative complication, conversion to open thoracotomy rate, and visual analog scale (VAS) scores on postoperative day (POD) 1 and VAS POD 2. Oncological outcomes were death from any cause at follow-up and lung cancer-related recurrence at 3 years. Postoperative complications were assessed from the day of surgery until the 30th POD or until hospital discharge if the postoperative hospital stay was longer than 30 days. The postoperative complications were evaluated by the Common Terminology Criteria for Adverse Events.^7)^ Conversion to thoracotomy was defined as either using a rib-spreader or lengthening the working incision beyond 8 cm. Number of lymph nodes retrieved was defined as the total number of lymph nodes dissected including mediastinal, subcarinal, and lobe-specific lymph nodes during the operation. The postoperative pain was recorded 1 and 3 days postoperatively using the VAS 0–10. Overall survival was defined as the time from the initial operation until death from any cause. Disease-free survival was defined as the time from the initial operation to the date of the lung cancer-related recurrence or progression (locoregional or distant).

### Data synthesis and analysis

Data were extracted based on demographic characteristics, postoperative complication, conversion to open thoracotomy rate, and VAS POD1 and VAS POD3. Data were collected using the Mantel–Haenszel random effects model with relative risk (RR) and mean difference (MD) as the effect size with a 95% confidence interval (CI). For analysis of survival, hazard ratios (HRs) and their standard errors (SEs) were pooled using the Inverse Variance random effect model. Statistical heterogeneity between groups was measured using the Higgins I^2^ statistic. Specifically, I^2^ = 0 indicates no heterogeneity, while we assume high heterogeneity based on an I^2^ value above 50%. Publication bias was assessed using a funnel plot. Analyses were performed using Review Manager 5.4.1. and *p*-value <0.05 was considered statistically significant.

Enhanced secondary analyses of survival curves were conducted, estimating individual patient time-to-event data extracted from published Kaplan–Meier (K-M) curves. The individual patient data (IPD), including event (mortality: 1; censored: 0) and time-to-cause (months), was estimated from Kaplan–Meier curves. We analyzed overall survival and disease-free survival. Data extraction followed the method outlined by Guyot et al.^8)^ Using WebPlotDigitizer, survival probabilities and corresponding time points were manually digitized from the K-M plots. The digitized data points, including time, survival probability, and number at risk, were used to estimate the individual patient survival times and censoring status. An R package designed for IPD reconstruction (e.g., IPDfromKM) was used to interpolate the extracted K-M data and create an approximate dataset representing each patient’s survival experience. The extracted data were used to reconstruct the underlying survival times for each patient, allowing for comprehensive survival analysis. K-M survival curves were generated for two treatment groups, MVATS and UVATS, and a log-rank test was performed to compare their survival distributions. We then fitted a Cox Proportional Hazards Model to estimate the HR for the UVATS group relative to the MVATS group, adjusting for potential confounders. The proportional hazards assumption was evaluated using Schoenfeld residuals and a global test, confirming the validity of the Cox model. Analysis was performed using R software 4.3.2.

The systematic quality of the included papers was evaluated using the recommended Newcastle–Ottawa Scale (NOS) for observational studies.^9)^ Investigations were classified as having low (<5 points), moderate (5–7 points), and high quality (>7 points).

## Results

### Study selection

**Fig. 1:**
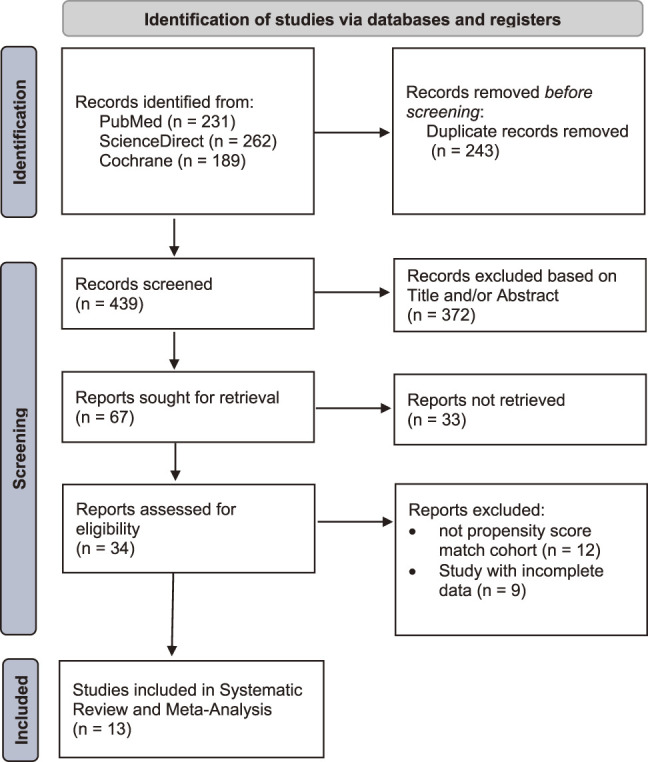
Selection and identification of studies with PRISMA guidelines. PRISMA: Preferred Reporting Items for Systematic Reviews and Meta-analyses

The search strategy identified relevant 682 studies to be included in the study. After screening titles and abstracts, 372 studies were excluded. A total of 34 studies underwent further assessments, 12 studies were excluded due to not propensity score match cohort, and 9 studies were excluded due to incomplete data present. A total of 13 studies were included and underwent further analysis. The flowchart of studies selection and identification are summarized in **Fig. 1**.

### Study characteristic

This study selected 13 propensity score match cohort studies consisting of 10 retrospective studies and 3 prospective studies which included 3180 patients. The characteristics of the studies are summarized in **Table 1**. The outcomes shown in this study were postoperative complication,^10–22)^ conversion to open thoracotomy rate^10,14,15,18,22)^ and VAS POD1,^11,13,17,21)^ VAS POD3,^11,13,17,21)^ and total lymph nodes retrieved.^11,13–17,20,22)^ There were 4 studies reporting survival curves for overall survival and disease-free survival.^16,17,19,20)^

**Table 1:** Characteristics of the studies analyzed

No.	Author	Years	Country	Cohort type	Surgery type	Number of cohort (after matched)		Age		Histopathology (adenocarcinoma) (%)	
						UVATS	MVATS	UVATS	MVATS	UVATS	MVATS
1	Bourdages- Pageau	2020	Canada	Retrospective	Lobectomy	247	247	65.7 ± 7.9	65.6 ± 7.8	75	76
2	Dai	2016	China	Retrospective	Lobectomy	63	63	58.68 ± 9.24	57.11 ± 12.22	85.71	80.95
3	Ke	2017	China	Retrospective	Lobectomy	40	40	59.3 ± 11.2	60.2 ± 11.7	NA	NA
4	Song	2017	Korea	Retrospective	Lobectomy	26	26	64.8 ± 9.7	65.0 ± 9.4	65.4	73.1
5	Shen	2016	China	Retrospective	Lobectomy	100	100	61.5 ± 7.9	60.9 ± 7.8	80	84
6	Ruan	2024	China	Retrospective	Lobectomy	106	106	60.54 ± 8.79	61.48 ± 10.68	96.2	95.2
7	Zhong	2021	China	Retrospective	Lobectomy	71	71	72.33 ± 3.7	73 ± 4.44	67.6	60.56
8	Mu	2015	China	Prospective	Lobectomy	47	47	56.67 ± 11.62	60.77 ± 11.04	77.6	88.8
					Wedge resection						
					Segmentectomy						
9	Sun	2022	China	Retrospective	Segmentectomy	143	143	54.6 ± 11.9	54.8 ± 13.7	NA	NA
10	Xie	2021	China	Retrospective	Segmentectomy	454	454	57.98 ± 10.52	57.98 ± 10.52	97.8	97.1
11	Chuang	2023	Taiwan	Prospective	Lobectomy	158	158	59.4 ± 10.5	60 ± 10.9	97.5	98.1
					Segmentectomy						
12	Homma	2022	Japan	Retrospective	Lobectomy	71	71	71.38 ± 8.97	70.52 ± 10.36	NA	NA
					Segmentectomy						
13	Dai	2023	China	Retrospective	Segmentectomy	64	64	50.41 ± 12.17	49.83 ± 12.10	100	100

UVATS: uniportal video-assisted thoracoscopic surgery; MVATS: multiportal video-assisted thoracoscopic surgery

### Risk of bias and study quality assessment

The risk of bias in the included studies was assessed using the NOS. The results of the bias assessment are summarized in **Table 2**. The included studies demonstrated moderate to good quality overall, as indicated by their NOS scores ranging from 6 to 8.

**Table 2:** The Newcastle—Ottawa Scale qualitative analysis of the included studies

Number	Author	Years	Selection				Comparability	Outcome			Total score
			1	2	3	4	1	1	2	3	
1	Bourdages- Pageau	2020	*	*	*	*	*	*	*	–	7
2	Dai	2016	*	*	*	*	*	*	*	*	7
3	Ke	2017	*	*	*	*	*	*	–	–	6
4	Song	2017	*	*	*	*	*	*	*	–	7
5	Shen	2016	*	*	*	*	*	*	*	–	7
6	Ruan	2024	*	*	*	*	*	*	*	*	8
7	Zhong	2021	*	*	*	*	*	*	*	*	8
8	Mu	2015	*	*	*	*	*	*	*	–	7
9	Sun	2022	*	*	*	*	*	*	*	–	7
10	Xie	2021	*	*	*	*	*	*	*	*	8
11	Chuang	2023	*	*	*	*	*	*	*	*	8
12	Homma	2022	*	*	*	*	*	*	–	–	6
13	Dai	2023	*	*	*	*	*	*	*	–	7

### Perioperative outcomes

**Fig. 2:**
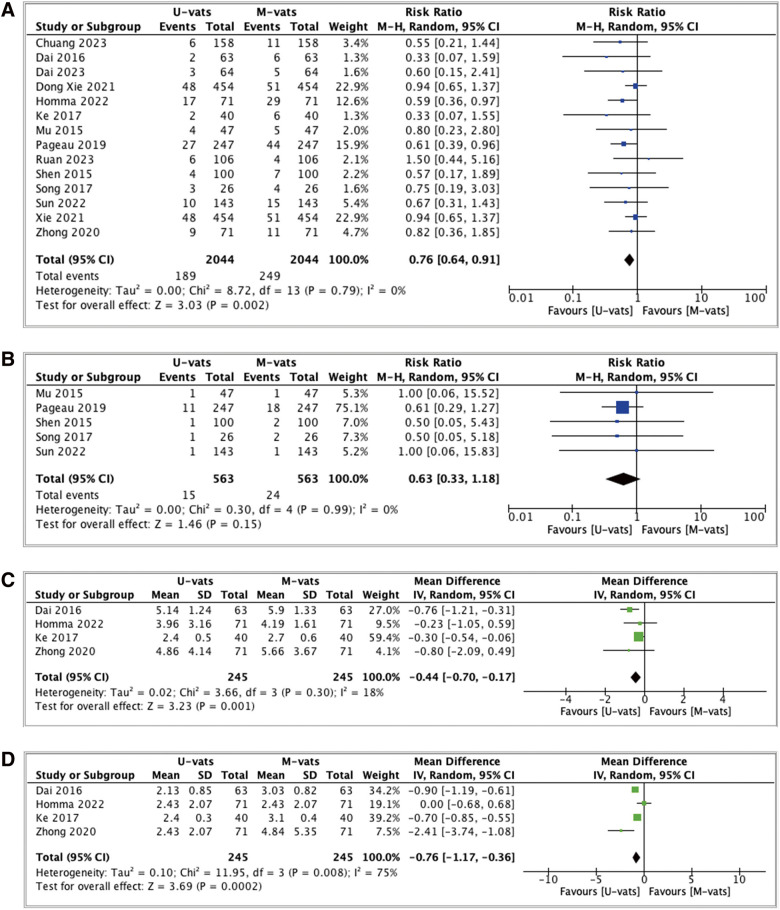
Forest plot of perioperative outcomes comparing UVATS and MVATS: (**A**) postoperative complications; (**B**) conversion to open thoracotomy; (**C**) VAS POD1; and (**D**) VAS POD3. UVATS: uniportal video-assisted thoracoscopic surgery, MVATS: multiportal video-assisted thoracoscopic surgery; VAS: visual analog scale [0–10], POD1: postoperative day 1, POD3: postoperative day 3

**Figure 2A** shows the outcome analysis of postoperative complications which compared uniport VATS with multiport VATS. Uniport VATS was associated with lower postoperative complication and the result was statistically significant (RR: 0.76; 95% CI: 0.64, 0.91; *p* = 0.002; I^2^ = 0%). In conversion to open thoracotomy, uniport VATS had a lower rate of conversion rather than multiport VATS but was not statistically significant (**Fig. 2B**, RR: 0.63; 95% CI: 0.33, 1.18; *p* = 0.15; I^2^ = 0%). Uniport VATS also showed better results compared to multiport VATS in pain management as shown by lower VAS POD1 and POD3 (MD: –0.44; 95% CI: –0.70, –0.17; *p* = 0.001; I^2^ = 18% and MD: –0.76; 95% CI: –1.17, –0.36; *p* <0.001; I^2^ = 75%, respectively) as shown in **Figs. 2C** and **2D**.

### Oncological outcomes

**Fig. 3:**
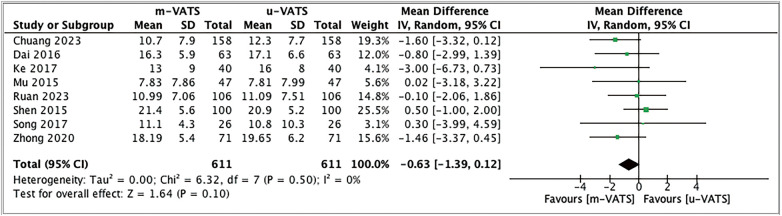
Forest plot mean difference with random effect models of total number lymph nodes retrieved using UVATS and MVATS in lobectomy and segmentectomy lung cancer. UVATS: uniportal video-assisted thoracoscopic surgery; MVATS: multiportal video-assisted thoracoscopic surgery

Total lymph nodes retrieved were higher in multiport VATS than uniport VATS (MD: –0.63, 95% CI: –1.39, 0.12, I^2^ = 0%, *p* = 0.1) as shown in **Fig. 3**. As this result was not statistically significant, uniport VATS may have comparable outcomes to multiport VATS.

### K-M overall survival analysis

**Fig. 4:**
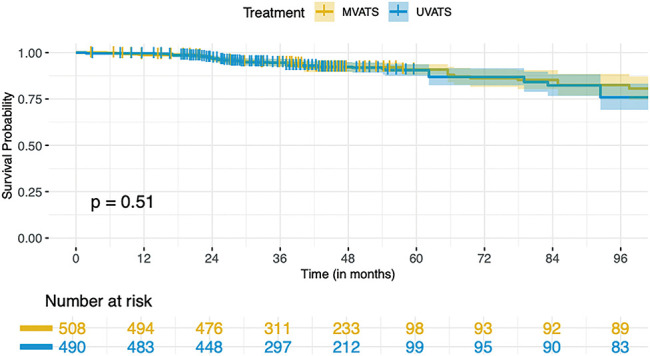
Kaplan–Meier survival curves comparing overall survival between UVATS and MVATS. UVATS: uniportal video-assisted thoracoscopic surgery; MVATS: multiportal video-assisted thoracoscopic surgery

**Fig. 5:**
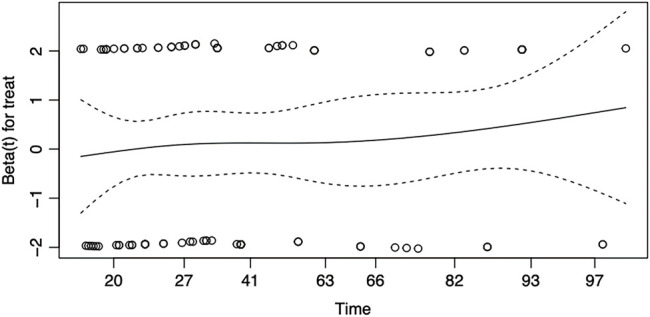
Schoenfeld residuals plot for the proportional hazards assumption in the Cox proportional hazards model for mortality comparing UVATS and MVATS. UVATS: uniportal video-assisted thoracoscopic surgery; MVATS: multiportal video-assisted thoracoscopic surgery

A pooled IPD meta-analysis from available K-M curves resulted in a total sample size of 998 patients from two treatment arms: MVATS (n = 508) and UVATS (n = 490). The survival probability at 96 months was slightly higher in the MVATS group (82.49%; 95% CI: 76.87%–88.52%) compared to the UVATS group (75.89%; 95% CI: 69.16%–83.29%) shown in **Fig. 4**. The log-rank test comparing survival between the MVATS and UVATS groups showed p-value of 0.5. This indicates no statistically significant difference in survival distributions between the two treatment groups over the entire follow-up period. The Cox model analysis revealed an HR of 1.1431 (95% CI: 0.7751–1.686) for the UVATS group compared to the MVATS group. The HR suggests a 14.31% higher risk of the event in the UVATS group, but this difference was not statistically significant (*p* = 0.5). The proportional hazards assumption was tested using Schoenfeld residuals and the global test. The global test yielded a p-value of 0.35, indicating no significant violation of the proportional hazards assumption. The Schoenfeld residuals plot showed no strong indication of violation, with the smoothed estimate remaining relatively flat over time and within the confidence intervals (**Fig. 5**).

#### K-M disease-free survival analysis

**Fig. 6:**
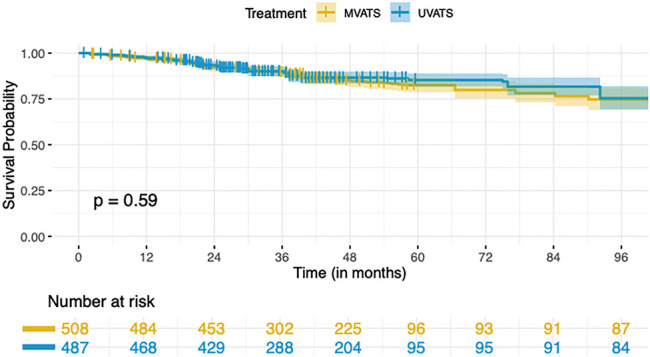
Kaplan–Meier survival curves comparing disease-free survival between UVATS and MVATS. UVATS: uniportal video-assisted thoracoscopic surgery; MVATS: multiportal video-assisted thoracoscopic surgery

**Fig. 7:**
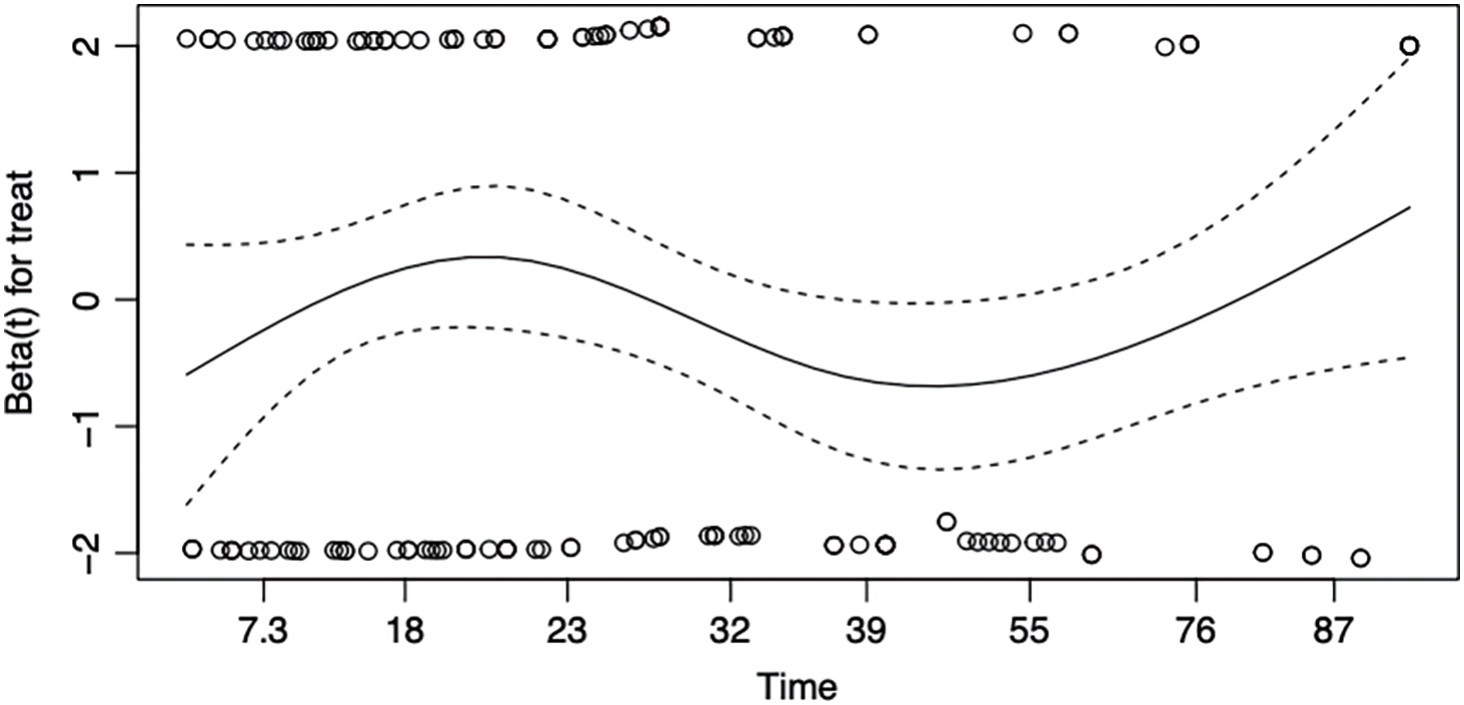
Schoenfeld residuals plot for the proportional hazards assumption in the Cox proportional hazards model for disease-free survival comparing UVATS and MVATS. UVATS: uniportal video-assisted thoracoscopic surgery; MVATS: multiportal video-assisted thoracoscopic surgery

A pooled IPD meta-analysis from available K-M curves resulted in a total sample size of 995 patients from two treatment arms: MVATS (n = 508) and UVATS (n = 487). The disease-free survival probability at 96 months was slightly higher in the UVATS group (75.43%; 95% CI: 69.36%–82.04%) compared to the MVATS group (74.74%; 95% CI: 69.04%–80.91%) shown in **Fig. 6**. However, the difference was not statistically significant (*p* = 0.59). The Cox model analysis revealed an HR of 0.9175 (95% CI: 0.6677–1.261) for the UVATS group compared to the MVATS group. The HR suggests an 8.25% lower risk of disease recurrence or failure in the UVATS group, but this difference was not statistically significant (*p* = 0.6). The proportional hazards assumption was tested using Schoenfeld residuals and the global test. The global test yielded a *p*-value of 1.00, indicating no significant violation of the proportional hazards assumption (**Fig. 7**).

## Discussion

This present meta-analysis sought not only perioperative outcomes but also oncological outcomes including disease-free survival and overall analysis for 3 years. This is the first meta-analysis to pool time-to-event data to obtain incidence of death and lung cancer-related recurrence. We conducted a meta-analysis of cohort studies that had performed propensity score matching, which aimed to equalize confounding factors. The studies included in this meta used 1:1 PSM. The use of PSM cohorts resulted in most of the data showing low heterogeneity. The heterogeneity of each outcome on the forest plot shows low heterogeneity (I^2^ <20%) as 0% in postoperative complications, 0% in conversion to thoracotomy rate, and 18% in VAS POD1, except for VAS POD3 heterogeneity is still high at 75%. Low heterogeneity means the proportion of total variation beyond chance across studies is low.

This present meta-analysis showed that the uniport VATS group had lower postoperative complications compared to multiport VATS (*p* = 0.002). In contrast to the randomized controlled study (RCT) study by Yao et al.,^23)^ there was no difference between the uniport VATS and multiport VATS groups in terms of postoperative complications. The uniport VATS group was also associated with a lower VAS POD1 compared to the multiport VATS group (*p* = 0.001). This is because, in the uniport VATS procedure, there is only one incision, resulting in less intercostal nerve injury.^11)^

Uniport VATS is performed with only one incision. Through this single incision, the thoracoscope and instruments are operated in one hole during surgery. This results in less trauma which results in avoiding severe damage to the blood vessels and intercostal nerves. Hence, postoperative pain is also less. Results from previous studies did suggest that uniport VATS provides better perioperative outcomes compared to multiport, but not all of them were significant. Some mentioned that the results are similar and feasible compared to multiport VATS,^14,21,22)^ and some mentioned that uniport VATS provides better benefits.^11,15)^ Unlike uniport VATS, multiport VATS requires 3 incisions. The first incision is for observation, the second is for the main surgical site, and the third is for sub-operation. There are many muscle layers in the back of the chest, as well as abundant blood vessels and nerve distribution. In addition, the intercostal space is also narrow, which allows bleeding during the incision.^24)^ Looking at short-term outcomes such as perioperative outcomes, this present meta-analysis shows that uniport VATS brings advantages with its less invasive technique and better short-term outcomes than multiport VATS.

In the context of oncology outcomes, this meta-analysis found that the number of retrieved lymph nodes was higher in the multiport VATS group although the results were not statistically significant (*p* = 0.1). The number of retrievable lymph nodes is an important parameter to evaluate whether radical surgery can be performed as a cancer treatment. It also serves as the basis for staging and postoperative prognosis. A combination of lobectomy and lymph node dissection is the standard surgical management for non-small-cell lung cancer (NSCLC).^25)^ According to the guidelines of the NCCN, it recommends the dissection of 3 or more mediastinal lymph nodes, and the total number of lymph nodes dissected is at least 12.^26)^ Meanwhile, guidelines from the European Society of Thoracic Surgeons recommend resection of at least 6 nodes to guarantee proper pathological classification.^27)^ In this meta-analysis, we only analyzed the number of lymph nodes retrieved, and the results were not statistically significant. This indicates that uniportal VATS is safe and feasible in lymph node dissection, although it cannot beat the superiority of multiport VATS.

Besides short-term outcomes, long-term prognosis is an important criterion to assess whether the uniport VATS approach is better than multiport VATS. Uniport VATS for lobectomy was first reported by Gonzalez-Rivaz in 2011,^28)^ while some hospital centers have only started applying it since 2014. Not many studies have reported on the long-term outcomes of uniport VATS such as overall survival and disease-free survival. This is the first meta-analysis comparing the long-term outcomes of uniport VATS with multiport VATS.

Despite the theoretical advantages of uniport VATS, such as reduced invasiveness and potentially faster recovery times, our analysis found no statistically significant differences between UVATS and MVATS in terms of both overall survival and disease-free survival over the 96-month follow-up period. The survival probability at 96 months was marginally higher in the UVATS group compared to the MVATS group for both overall survival and disease-free survival. However, the differences were not statistically significant, as indicated by the p-values of 0.5 for overall survival and 0.59 for disease-free survival. This lack of significant difference was further supported by the Cox proportional hazards models, which showed HRs close to 1 for both outcomes, suggesting that the risk of mortality and disease recurrence or failure is comparable between the 2 surgical approaches.

The choice between UVATS and MVATS may be guided more by surgeon experience and patient preference rather than by a substantial difference in long-term outcomes. While UVATS has been promoted for its potential benefits, including better postoperative pain management and quicker recovery, our results do not demonstrate a survival advantage for UVATS over MVATS in the context of lobectomy and segmentectomy for lung cancer. This suggests that both techniques are equally effective from a long-term oncological perspective. It is also important to note that the proportional hazards assumption was tested and confirmed to be valid for both overall survival and disease-free survival, reinforcing the reliability of the Cox model results. The Schoenfeld residual plots showed no significant deviation, further supporting the conclusion that the HRs reported are stable over time. The non-significant differences observed in this study could be attributed to the advances in both UVATS and MVATS techniques, which have been refined over the years to minimize surgical trauma while ensuring effective oncological resection. Additionally, the lack of significant differences may reflect the importance of other factors, such as tumor biology, patient comorbidities, and adjuvant therapies, which can heavily influence long-term outcomes irrespective of the surgical approach.

This study acknowledges a few limitations. First, a small number of cohort studies provided long-term oncological outcomes comparing uniport VATS and multiport VATS in lung cancer management. All included studies were cohort studies. While these studies were generally moderate-to-high quality, they did not substitute for large-scale RCT.

## Conclusion

This present meta-analysis concluded perioperative outcomes were more beneficial using uniport VATS. Uniport VATS can prove its clinical effectiveness and is similar to multiport VATS. In conclusion, our study suggests that both UVATS and MVATS are viable options for the surgical management of lobectomy and segmentectomy in lung cancer, offering comparable long-term outcomes in terms of overall and disease-free survival. The choice of surgical technique should be individualized based on patient factors, surgeon expertise, and available resources. Further studies with larger sample sizes and longer follow-up periods may help to elucidate any subtle differences between these approaches, particularly in specific patient subgroups.

## Declarations

### Ethics approval and consent to participate

Not applicable.

### Consent for publication

Not applicable.

### Funding

The authors receive no financial support for the research authorship and publication.

### Author contributions

Conception and design: IWS

Analysis and interpretation: IWS and PFKP

Data collection: KPY and IKAPH

Writing the article: IKAPH and PFKP

Critical revision of the article: IWS and KPY

Final approval of the article: IWS, PFKP, KPY, and IKAPH.

### Disclosure statement

The authors report no conflicts of interest.
